# Supplementary figures and images for: Fitness models provide accurate short-term forecasts of SARS-CoV-2 variant frequency
Source: PLoS Comput Biol. 2024 Sep 6;20(9):e1012443. doi: 10.1371/journal.pcbi.1012443 (PMC11410224; doi:10.1371/journal.pcbi.1012443)

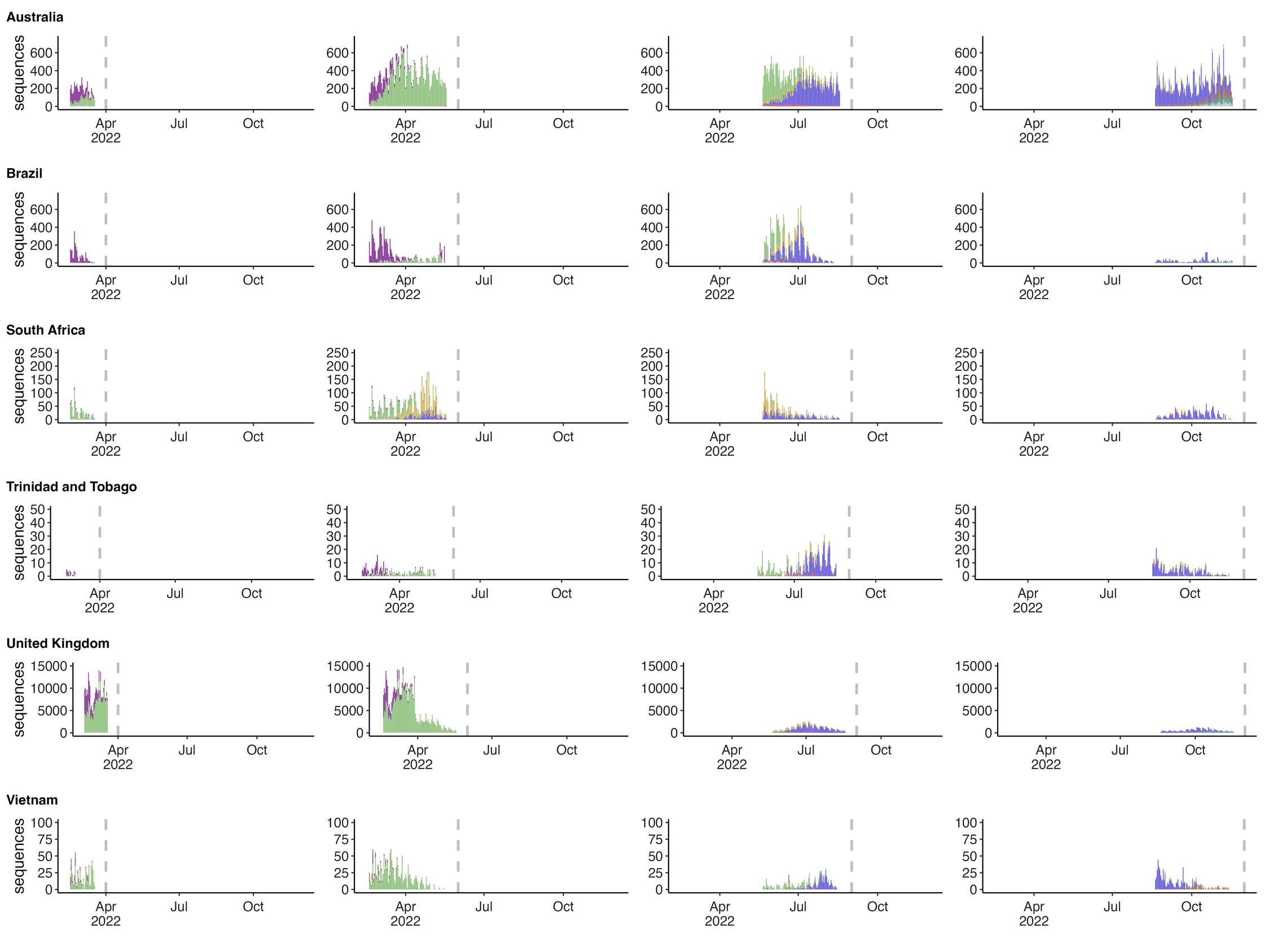

Supplement: S1 Fig — (A) Variant sequence counts categorized by Nextstrain clade at 4 different analysis dates. (TIF) [file pcbi.1012443.s001.tif]

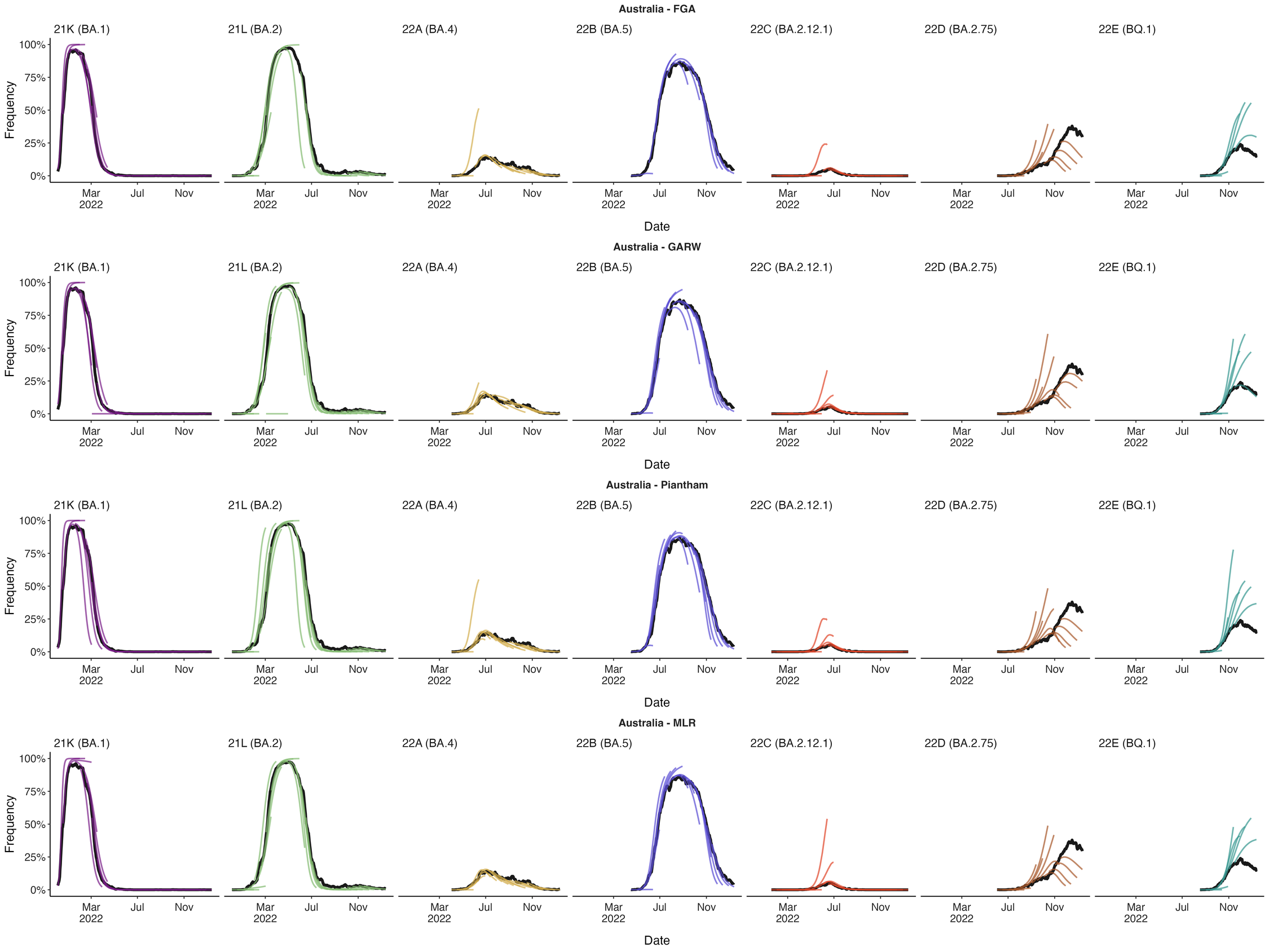

Supplement: S2 Fig — (A) +30 day frequency forecasts for variants in bimonthly intervals using the MLR model for Australia. Each forecast trajectory is shown as a different colored line. Retrospective smoothed frequency is shown as a thick black line. (TIF) [file pcbi.1012443.s002.tif]

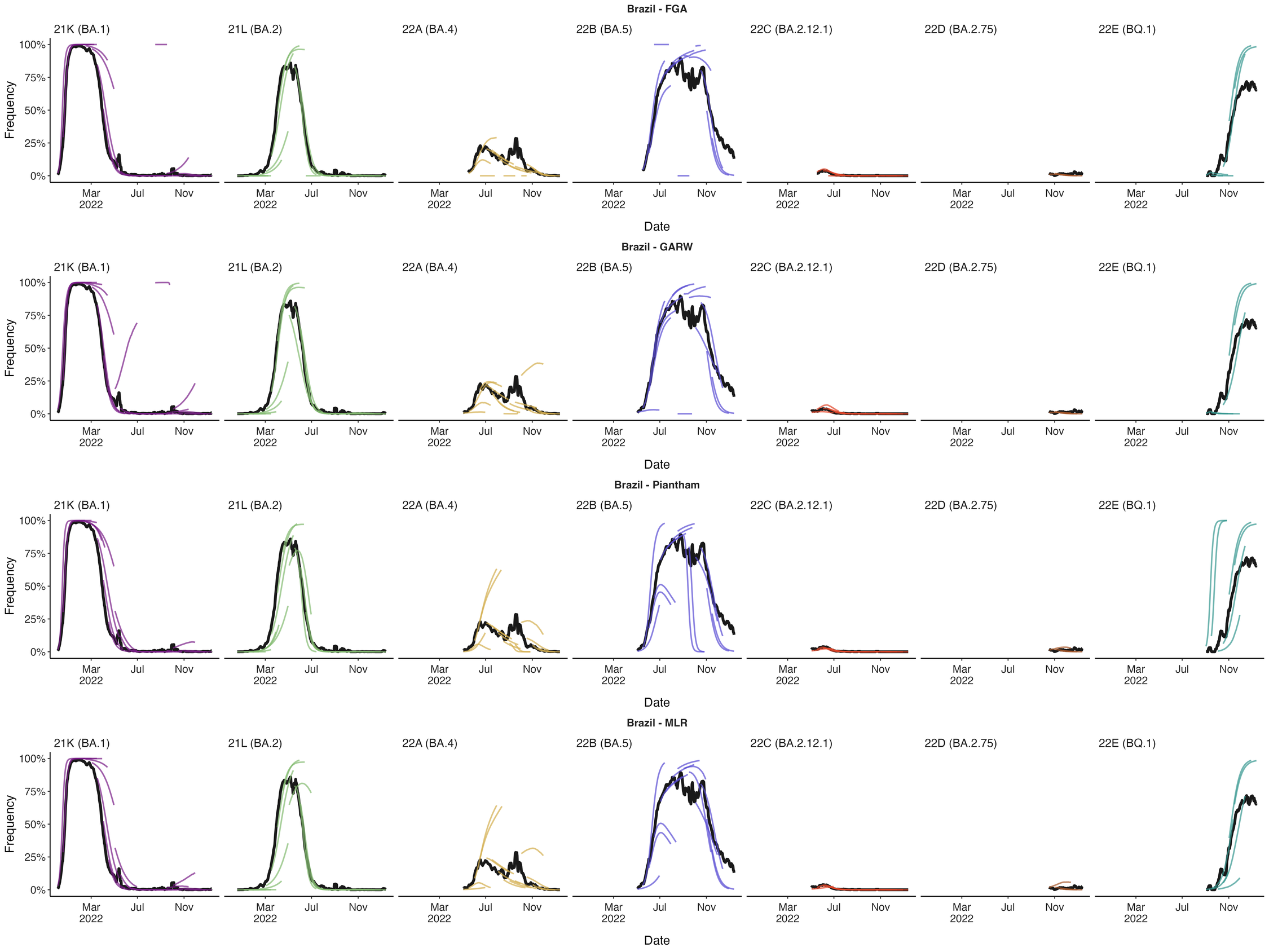

Supplement: S3 Fig — (A) +30 day frequency forecasts for variants in bimonthly intervals using the MLR model for Brazil. Each forecast trajectory is shown as a different colored line. Retrospective smoothed frequency is shown as a thick black line. (TIF) [file pcbi.1012443.s003.tif]

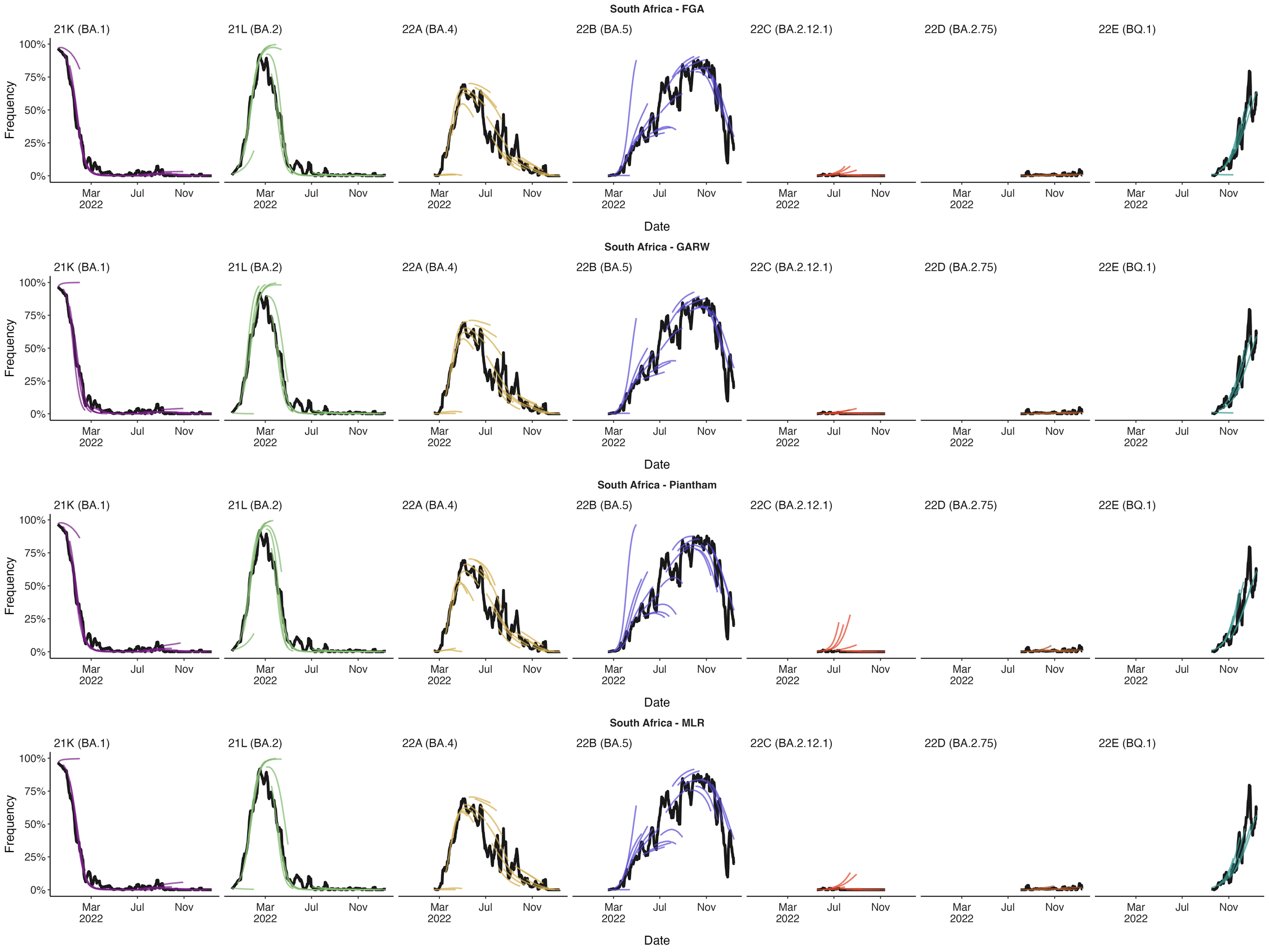

Supplement: S4 Fig — (A) +30 day frequency forecasts for variants in bimonthly intervals using the MLR model for South Africa. Each forecast trajectory is shown as a different colored line. Retrospective smoothed frequency is shown as a thick black line. (TIF) [file pcbi.1012443.s004.tif]

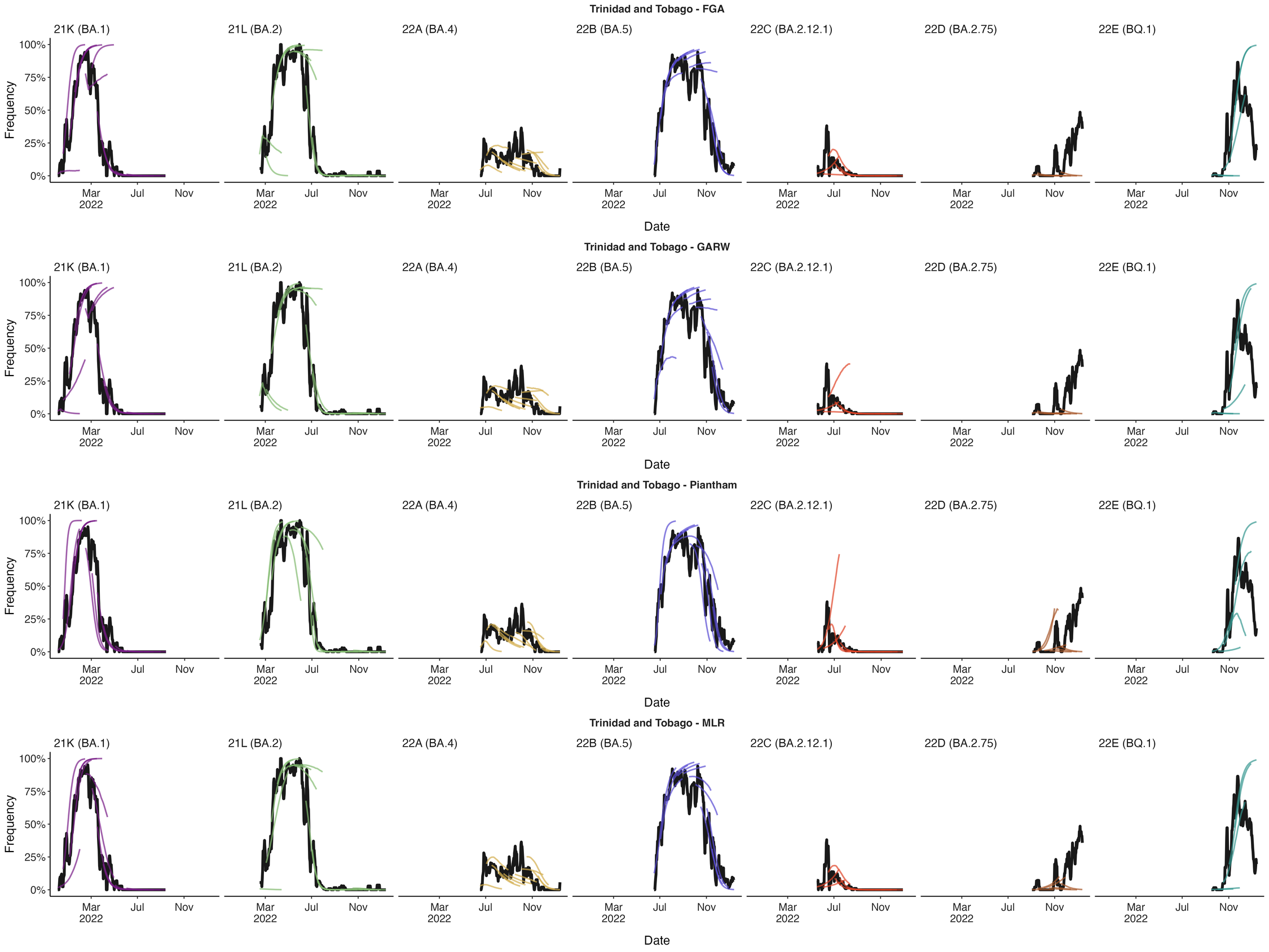

Supplement: S5 Fig — (A) +30 day frequency forecasts for variants in bimonthly intervals using the MLR model for Trinidad and Tobago. Each forecast trajectory is shown as a different colored line. Retrospective smoothed frequency is shown as a thick black line. (TIF) [file pcbi.1012443.s005.tif]

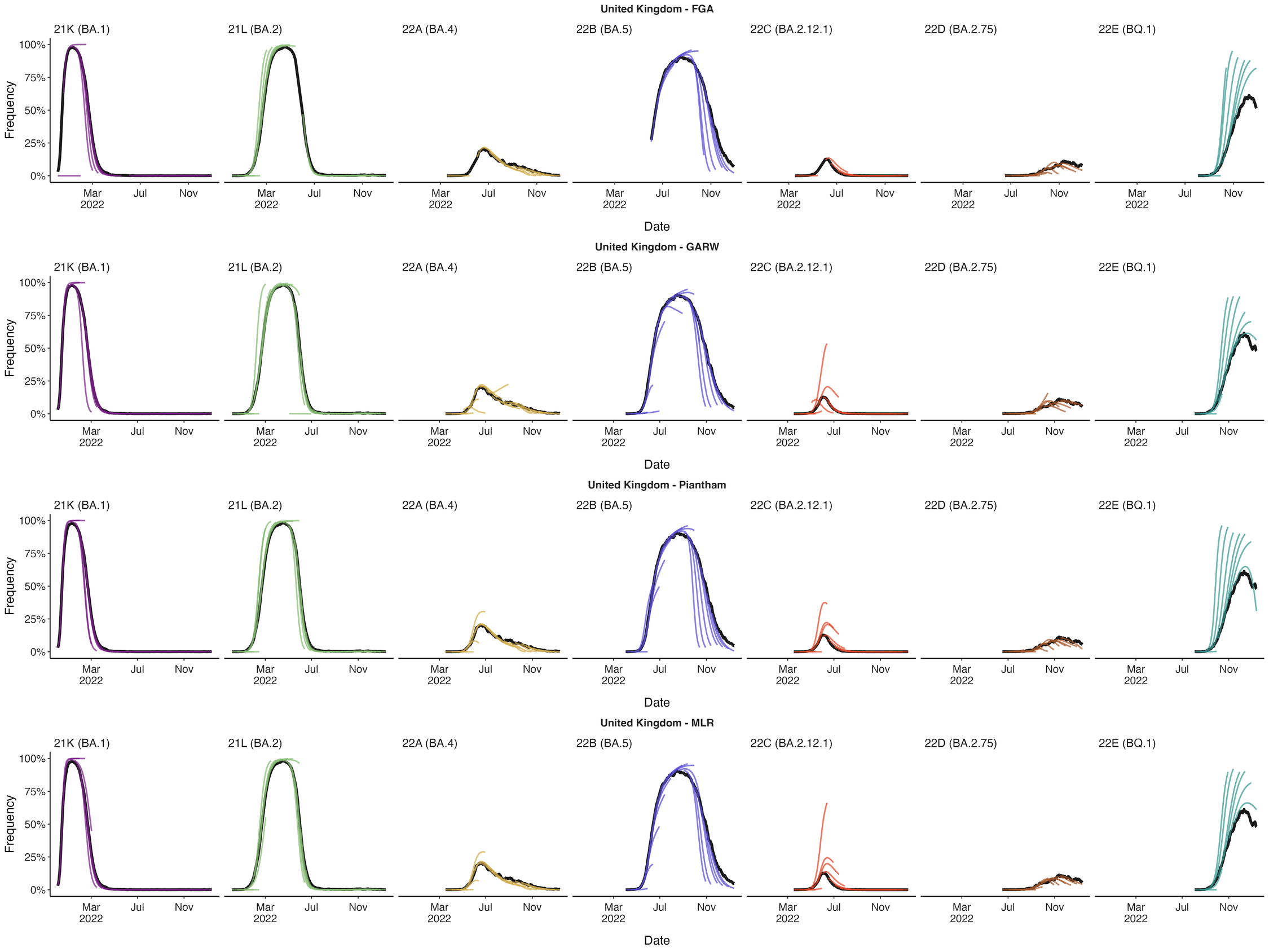

Supplement: S6 Fig — (A) +30 day frequency forecasts for variants in bimonthly intervals using the MLR model for United Kingdom. Each forecast trajectory is shown as a different colored line. Retrospective smoothed frequency is shown as a thick black line. (TIF) [file pcbi.1012443.s006.tif]

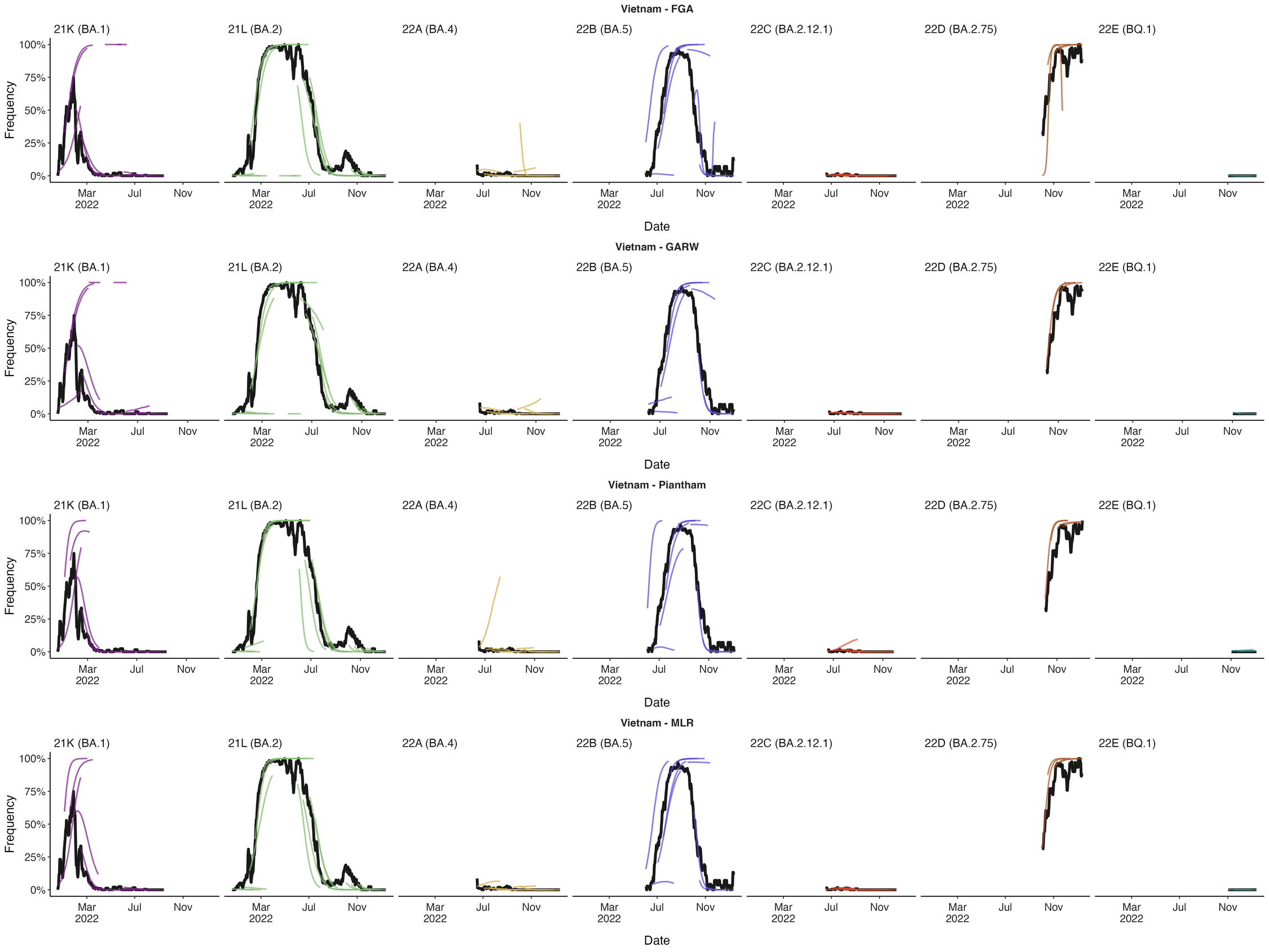

Supplement: S7 Fig — (A) +30 day frequency forecasts for variants in bimonthly intervals using the MLR model for Vietnam. Each forecast trajectory is shown as a different colored line. Retrospective smoothed frequency is shown as a thick black line. (TIF) [file pcbi.1012443.s007.tif]

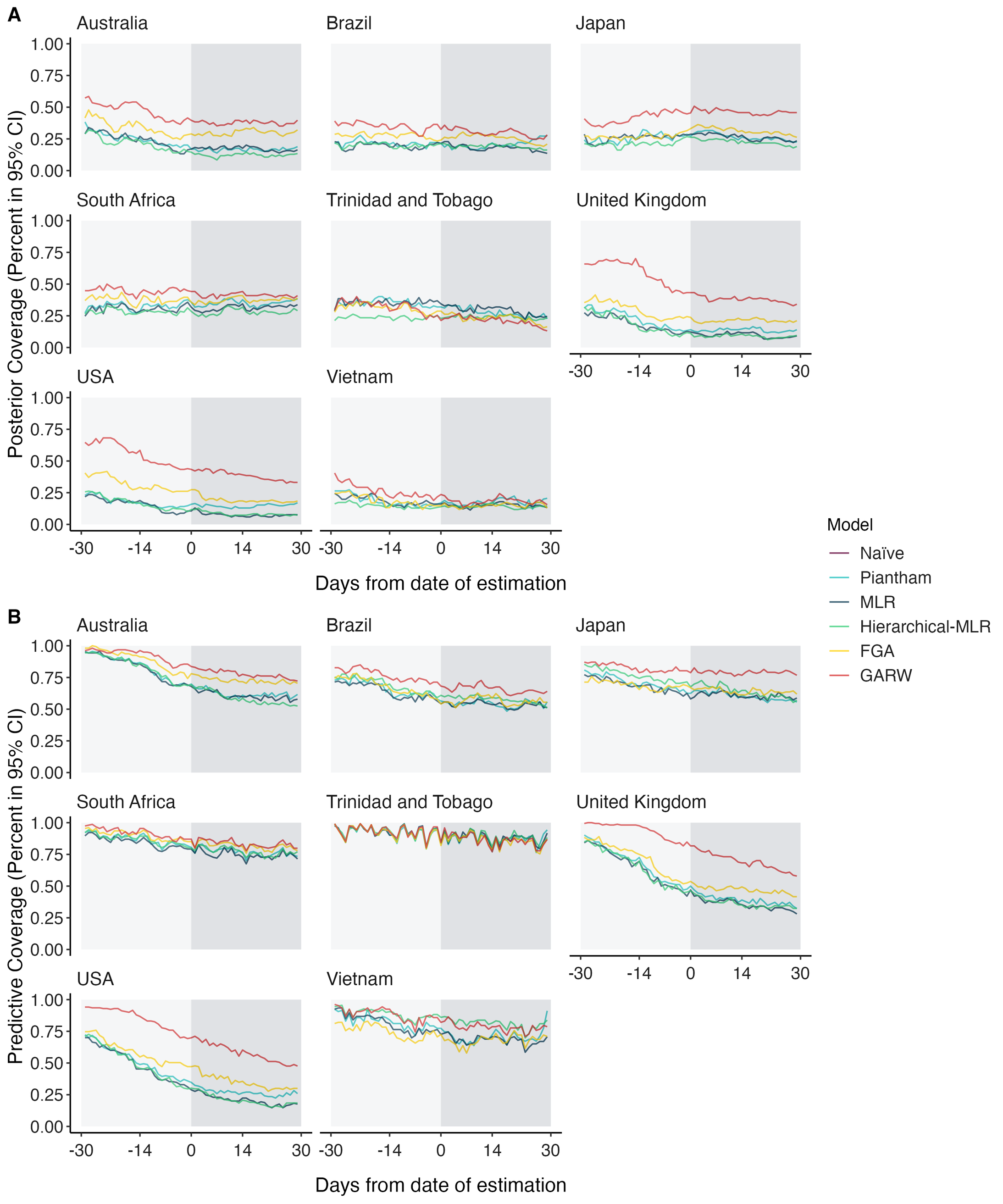

Supplement: S8 Fig — (A) The proportion of estimates lying within the 95% confidence intervals (CIs) of posterior latent frequencies across lag times (-30,-30). (B) The proportion of estimates lying within the 95% confidence intervals (CIs) of posterior predictive sample frequencies across lag times (-30,-30). We generate the posterior predictive sample frequencies by sampling random counts for each variant using their posterior latent frequencies conditioning on the total sequences being those observed retrospectively. (TIF) [file pcbi.1012443.s008.tif]

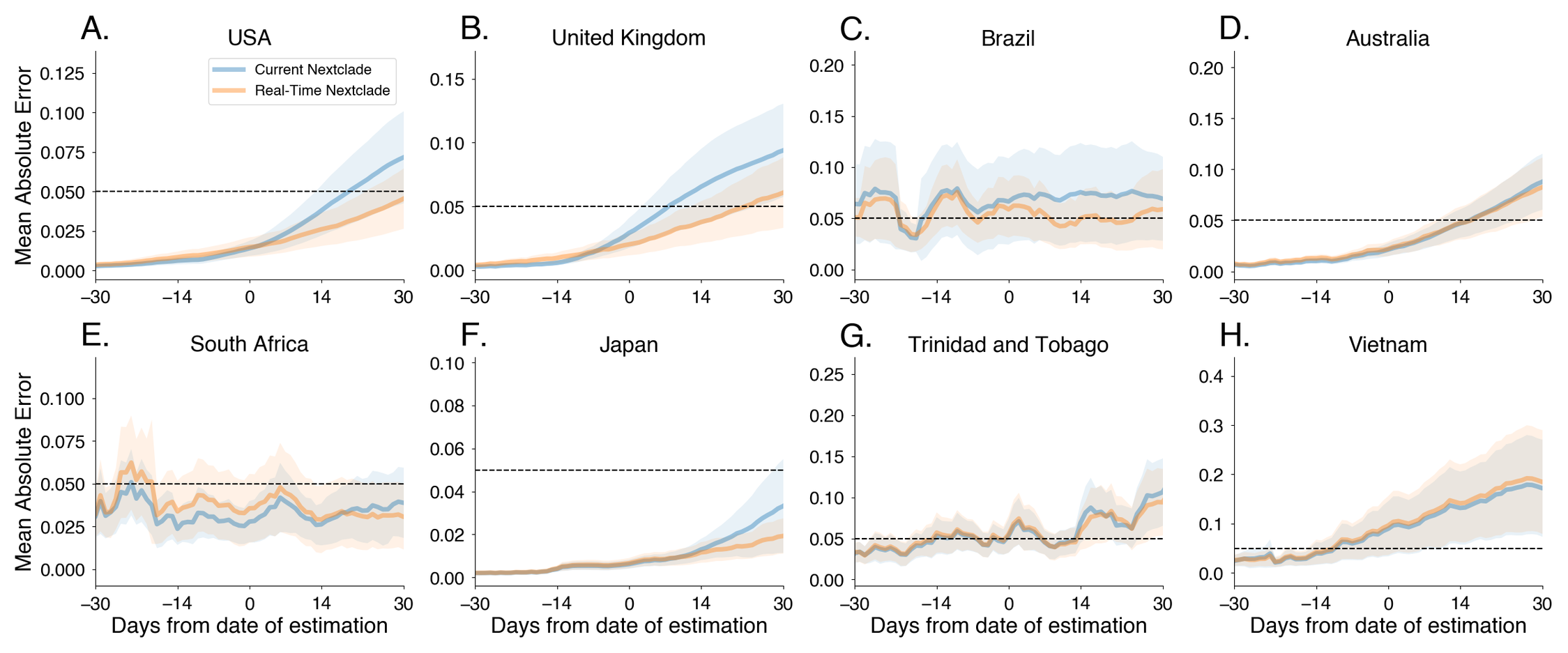

Supplement: S9 Fig — (A-H) Mean absolute error for MLR as a function of days since date of estimation, starting from 30 day hindcasts to 30 days forecasts. Intervals shown have width of two standard errors of the mean. We compare retrospective Nextstrain clade assignments made today (‘Current Nextclade’) to Nextstrain clade assignments available in Oct 2022 (‘Real- time Nextclade’). We find that errors are qualitatively similar regardless of Nextclade version with errors being potentially higher for the current Nextclade version. (TIF) [file pcbi.1012443.s009.tif]

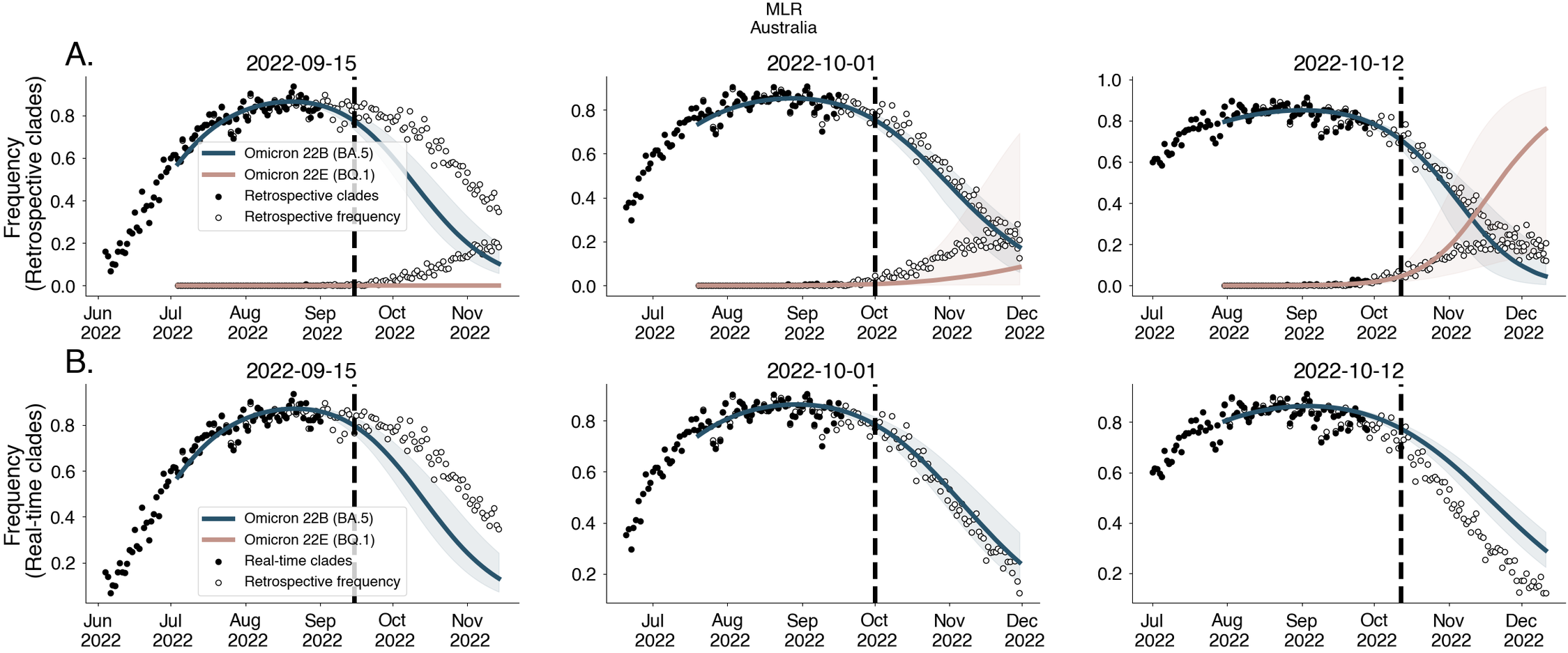

Supplement: S10 Fig — Forecasts from MLR fit to data generated using retrospective Nextstrain clade designations (‘Current Nextclade’) (A) and Nextstrain clade assignments available in Oct 2022 (‘Real-time Nextclade’) (B). (TIF) [file pcbi.1012443.s010.tif]

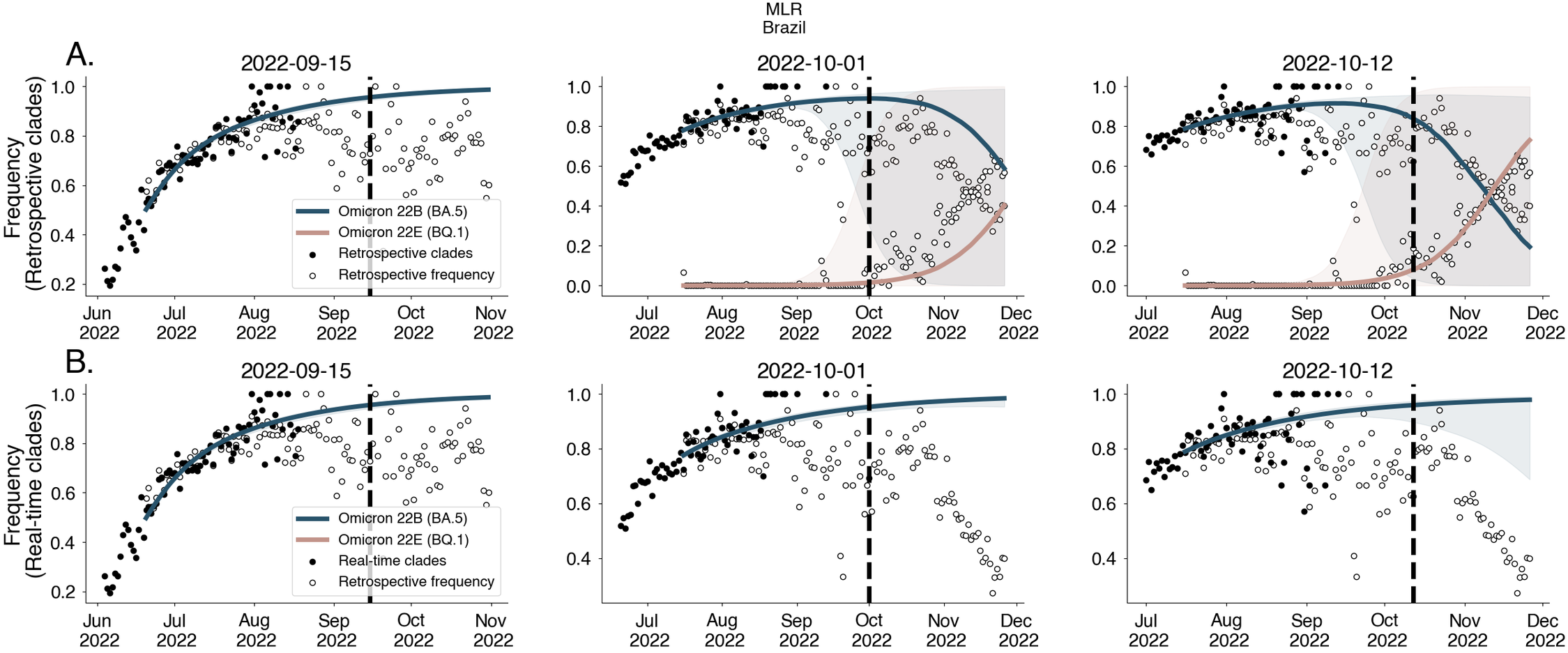

Supplement: S11 Fig — Forecasts from MLR fit to data generated using retrospective Nextstrain clade designations (‘Current Nextclade’) (A) and Nextstrain clade assignments available in Oct 2022 (‘Real-time Nextclade’) (B). (TIF) [file pcbi.1012443.s011.tif]

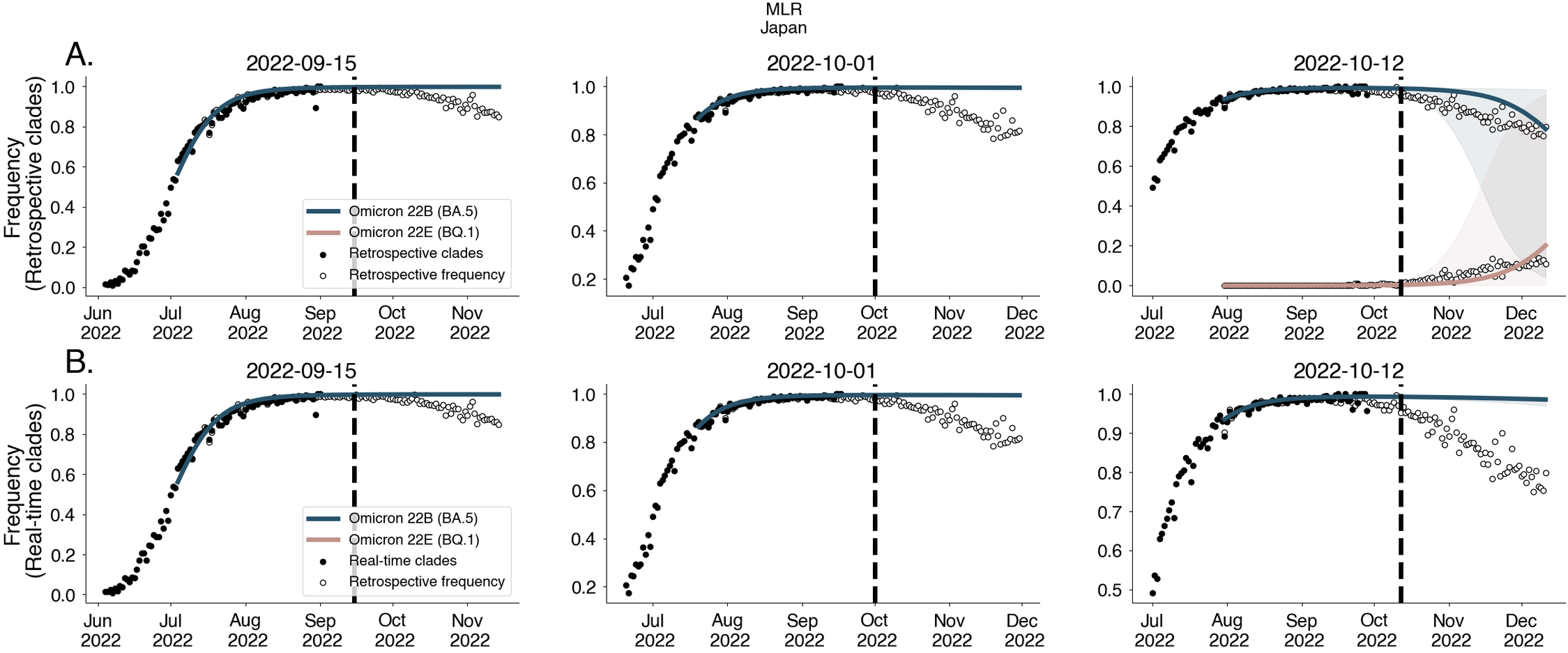

Supplement: S12 Fig — Forecasts from MLR fit to data generated using retrospective Nextstrain clade designations (‘Current Nextclade’) (A) and Nextstrain clade assignments available in Oct 2022 (‘Real-time Nextclade’) (B). (TIF) [file pcbi.1012443.s012.tif]

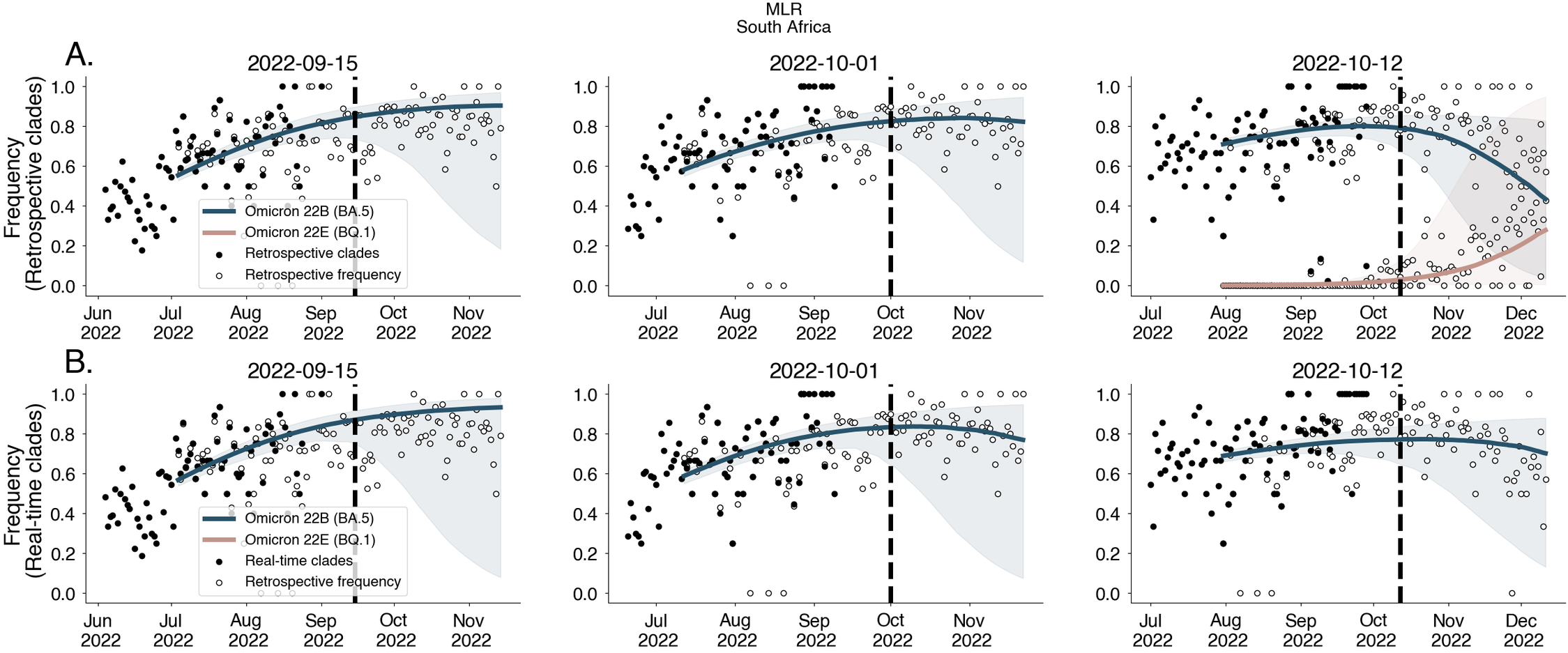

Supplement: S13 Fig — Forecasts from MLR fit to data generated using retrospective Nextstrain clade designations (‘Current Nextclade’) (A) and Nextstrain clade assignments available in Oct 2022 (‘Real-time Nextclade’) (B). (TIF) [file pcbi.1012443.s013.tif]

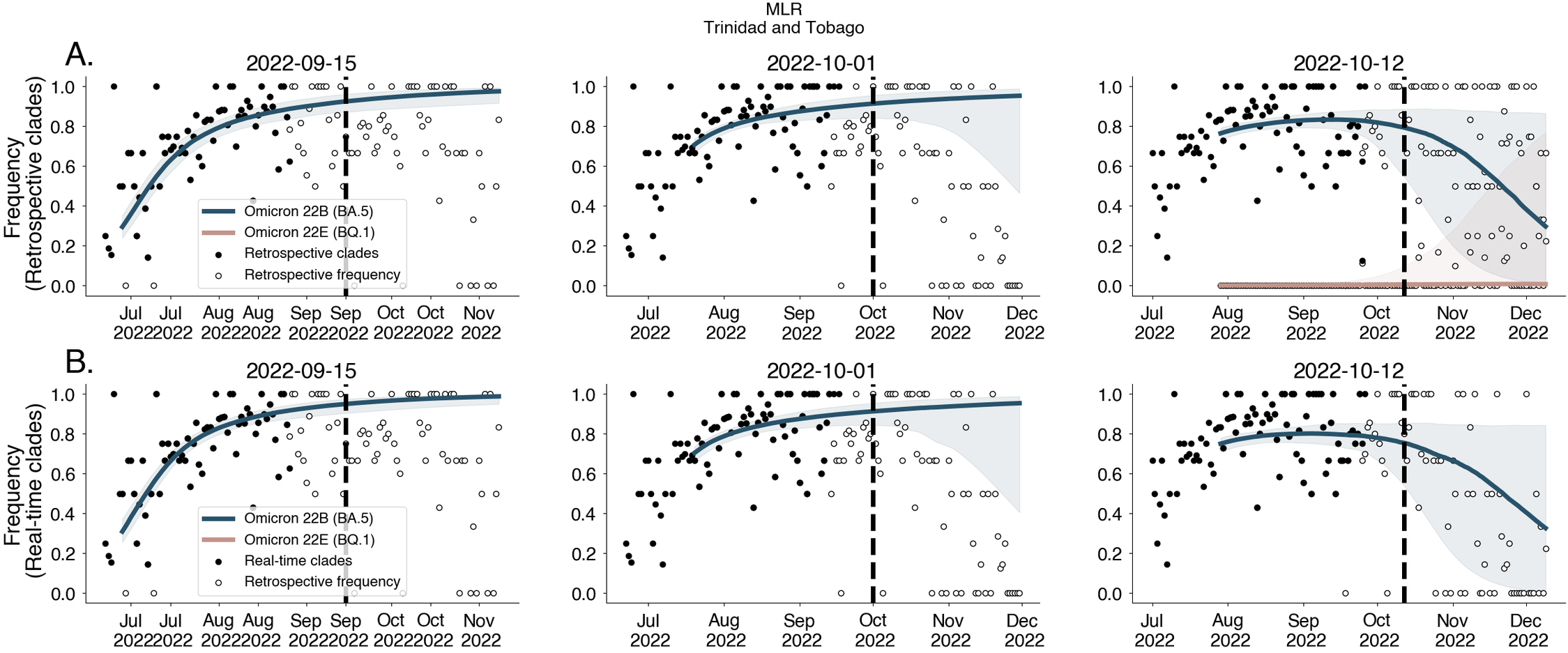

Supplement: S14 Fig — Forecasts from MLR fit to data generated using retrospective Nextstrain clade designations (‘Current Nextclade’) (A) and Nextstrain clade assign- ments available in Oct 2022 (‘Real-time Nextclade’) (B). (TIF) [file pcbi.1012443.s014.tif]

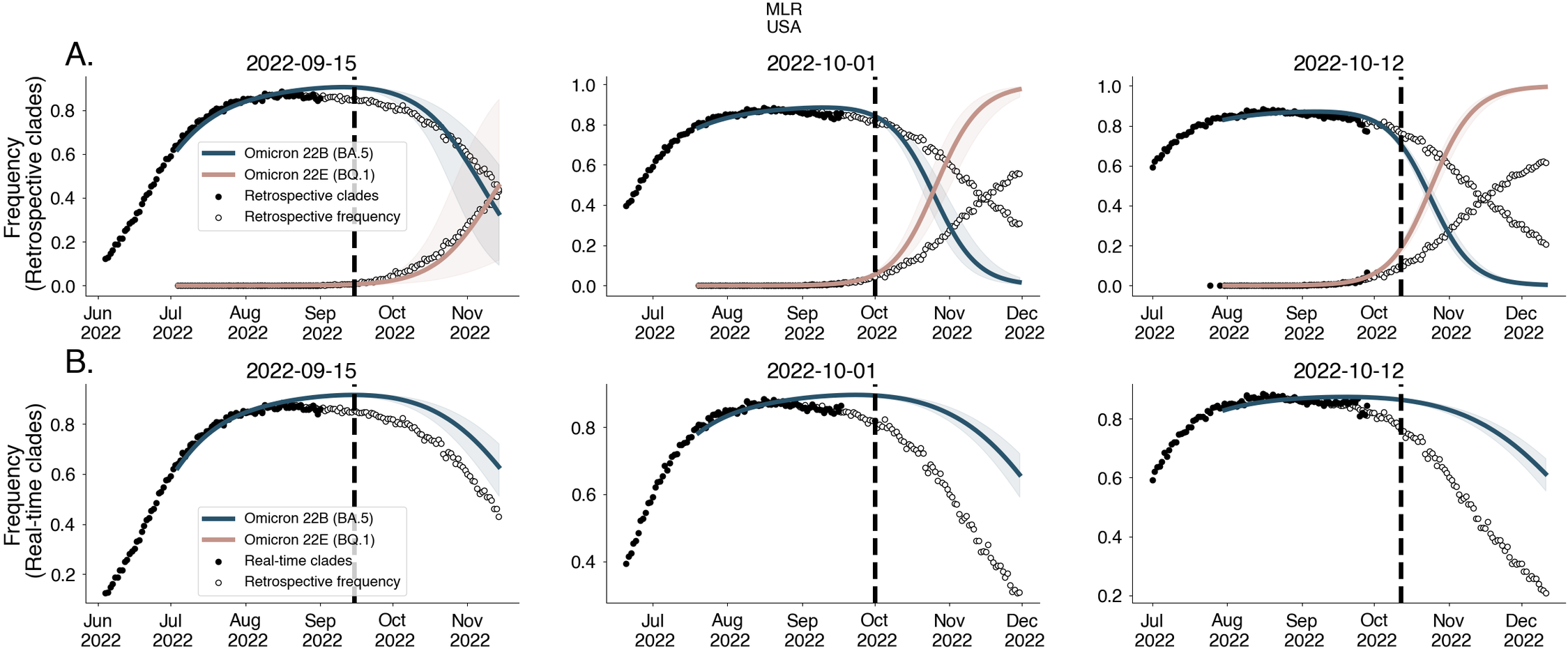

Supplement: S15 Fig — Forecasts from MLR fit to data generated using retrospective Nextstrain clade designations (‘Current Nextclade’) (A) and Nextstrain clade assignments available in Oct 2022 (‘Real-time Nextclade’) (B). (TIF) [file pcbi.1012443.s015.tif]

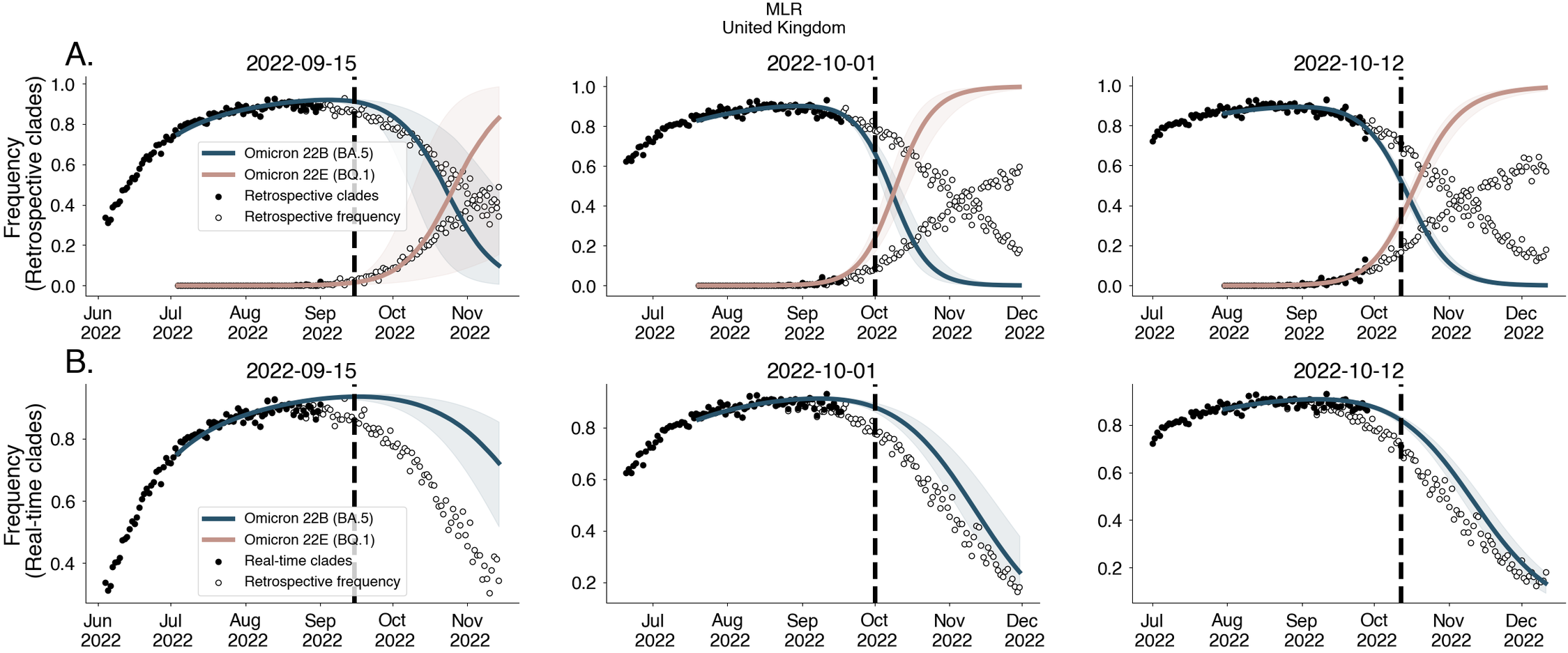

Supplement: S16 Fig — Forecasts from MLR fit to data generated using retrospective Nextstrain clade designations (‘Current Nextclade’) (A) and Nextstrain clade assignments available in Oct 2022 (‘Real-time Nextclade’) (B). (TIF) [file pcbi.1012443.s016.tif]

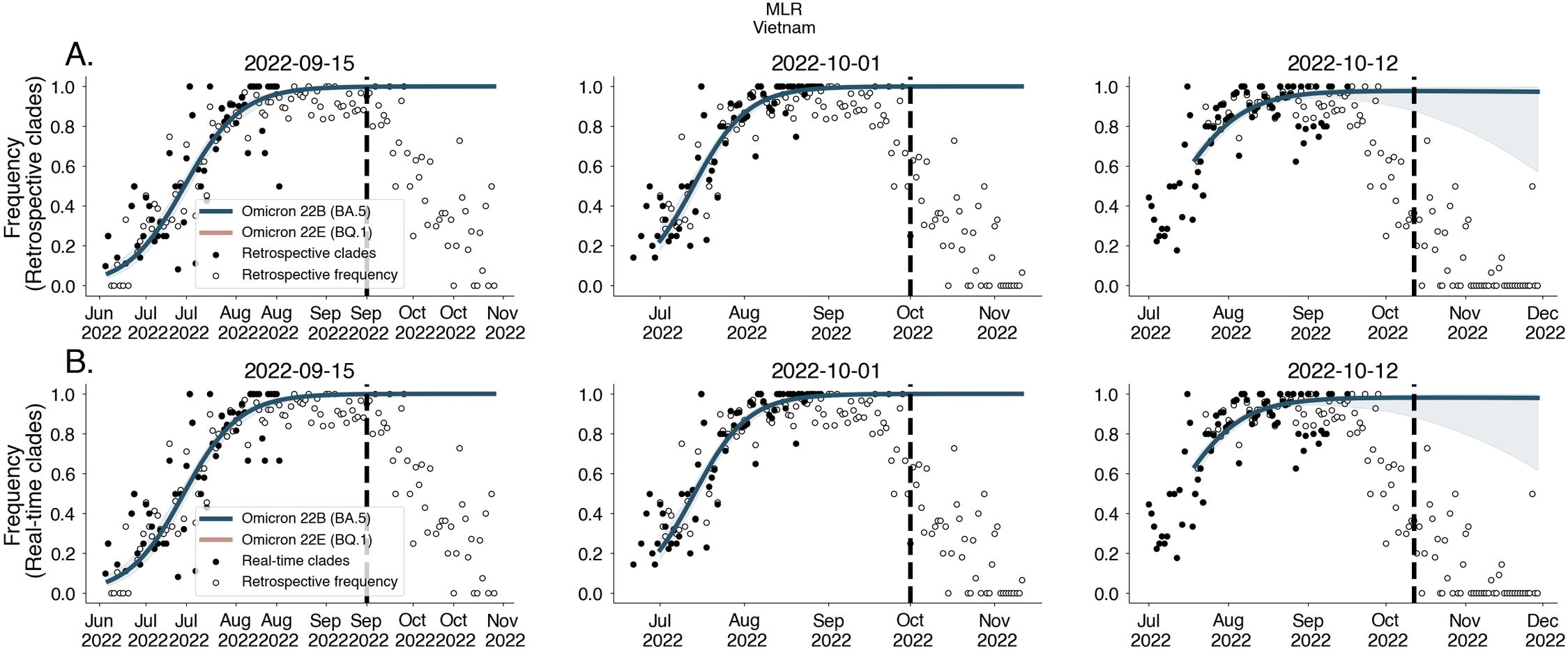

Supplement: S17 Fig — Forecasts from MLR fit to data generated using retrospective Nextstrain clade designations (‘Current Nextclade’) (A) and Nextstrain clade assignments available in Oct 2022 (‘Real-time Nextclade’) (B). (TIF) [file pcbi.1012443.s017.tif]
