# Supplementary material for: Fitness models provide accurate short-term forecasts of SARS-CoV-2 variant frequency
Source: PLoS Comput Biol. 2024 Sep 6;20(9):e1012443. doi: 10.1371/journal.pcbi.1012443 (PMC11410224; doi:10.1371/journal.pcbi.1012443)
Supplement: Attachment — Submitted filename: Manuscript Review Response.pdf [file pcbi.1012443.s019.pdf]

Fitness models provide accurate short-term forecasts of SARS-CoV-2 variant frequency

PLOS Computational Biology Response Letter

Dear Editor,

We appreciate your expertise and time in reviewing our paper and providing valuable comments. It was your valuable and insightful comments that led to possible improvements in the current version. We have carefully considered the comments and tried our best to address each of them. Below, we've included point-by-point responses to reviewer critiques.

Sincerely,

Eslam Abousamra, Marlin Figgins, and Trevor Bedford

Reviewer 1:

In this manuscript, the authors perform a retrospective analysis to compare the performance of 4 different models aimed at nowcasting and forecasting variant frequency. They construct reference data sets to mimic the SARS-CoV-2 sequences that would have been available in GISAID at each reference date in 6 different countries, and apply a single Nextstrain clade assignment model across all reference datasets. The Nextstrain clade assignment categorizes each sequence into one of 7 clade assignments that correspond to the circulating and emerging lineages in 2022: BA.1, BA.2, BA.4, BA.5, BA.2.12.1, BA.2.75, and BQ.1. They then pass these datasets of sequence counts by collection date into each of the 4 models and a baseline "naïve" model for comparison, for each country and each reference date. They assess the performance of each model using median and mean absolute error in the variant frequency estimate of each model, where ground truth is defined as the 7-day rolling averaged variant proportion from a later reference date (when presumably all/most sequences collected on those days had been submitted). They average model performance across all variants and reference time points and examine how model performance varies from 30 days before the forecast time (nowcast) to 30 days ahead of the forecast time. As a secondary analysis, they investigate the relationship between sequence quantity and quality on model performance, both empirically (by comparing across the countries with differences in sequencing characteristics) and through simulation. They generate simulated datasets with reduced sequencing levels by randomly sampling from the available sequences to obtain a constant number of sequences per week. They then refit the models to each of the datasets at the different sequencing levels and compare the average performance of the models.

The authors acknowledge that while the multinomial logistic regression (MLR) model performs "well" when averaging over all variants and reference time points, a subset of predictions have very large error. They hypothesize that this error is largest when variants are emerging and there are comparably few sequences of that variant.

My review:

The authors have addressed a very critical problem in the field of public health, particularly relevant in the wake of an explosion in the availability of genomic

sequencing and its use for public health decision-making for COVID-19. While a number of models have been developed, and a number of estimates of variant frequencies are made public, there is comparably little rigorous evaluation of the nowcasts and forecasts of these model outputs. One of the reasons evaluation is less straightforward than evaluation of forecasts of other epidemiological indicators such as hospital admissions, cases, or deaths, is that the definition of the quantity of interest itself is also dynamic – the upstream models used to assign a sequence to a particular lineage are themselves constantly changing. Acknowledging this difficulty and complexity of the problem the authors tried to tackle, I still have a number of reservations about the work:

1 – The authors describe how they attempted to reconstruct datasets that would have been available as of each reference date, using only sequences that had been submitted by a certain date. This made sense, however, it seems that they applied the same Nextstrain clade assignment model across all of the reference datasets. If the Nextstrain clade assignment model is at all similar to pangolin assignment model, the assignments are not stable over time. Therefore, to do this kind of evaluation properly you would need to have run the clade assignment using the Nextstrain clade assignment model available at the time that the estimate was made, and then compare it to the observed proportions based on the updated clade assignment model, because this represents what you would have forecasted with the information available at the time. For this reason, I don't think the authors can claim that this analysis is truly "real-time" evaluation, but I do understand why they made this choice as it makes for a clean comparison if purely concerned with model performance given a stable set of lineage assignment information.

We thank the reviewer for pointing out this important distinction. We have performed an additional analysis to address this concern of clade designation dynamics specifically for an emerging variant (clade 22E / lineage BQ.1) in October 2022. Here directly compared datasets available at the time that didn't yet have clade 22E broken out as its own separate clade to retrospective datasets that break out clade 22E (where both scenarios use the same sequences, just different clade designations). These results are

shown in new Supp. Figure S9. We additionally show differences in real-time forecasts and retrospective forecasts in new Supp. Figures S10 through S17. We describe these findings in new subsection in Methods titled “Comparing forecasts using retrospective clade designations and real-time designations”:

> *“The main analyses discussed in this manuscript rely on subsetting and filtering SARS-CoV-2 sequence metadata accessed on a particular data. However, the clade designations used throughout this manuscript may not have been the same as clade designations at the time the data was available. To understand how this affects our evaluation of forecast error, we compare the accuracy of models fit to the sequence counts from metadata at the time and using the available Nextclade version to those fit on the retrospective Nextclade version used in the rest of the analyses in this paper. In particular, we focus on the timing of the designation of Omicron 22E in October 2022 and show the accuracy of MLR using the different data sets at different forecast leads. We compare the resulting MAE of these analyses between Nextclade versions in Supp. Figure (S9) and show trajectories from individual countries in Supp. Figures (S10-S17)”*

2 – The authors did not include any incorporation of the uncertainty of estimates into their evaluation. Given the stated hypothesis that models perform worse when there is limited data about emerging variants, it feels critical to evaluate model estimates with uncertainty, rewarding models who properly quantify the uncertainty at those critical times. Even if not every model returns a full distribution of estimates, they could at the least do an analysis using the confidence intervals of the estimates and assessing the coverage of their intervals compared to the “truth” data

We agree that the incorporation of uncertainty estimates is indeed crucial. We have made revisions to incorporate a coverage metric to our framework and include a figure that shows the proportion of estimates lying within 95% CI for both the posterior and predictive coverage (Supp. Fig. 8). We observe decreasing coverage going from –30

days to +30 days from the lead date. Additionally, we have included “Assessing coverage for short-term frequency forecasts” sub-section in the Methods section:

> *“The main results of our analyses rely on mean and median absolute error as metrics, however, there is much to gain by using probabilistic forecasts for variant frequency. To this aim, we investigate the coverage of these different methods for forecasting variant frequency. Though not all models described initially were designed with uncertainty quantification in mind, we develop and fit Bayesian extensions of these models which are fit to the same data sets as before using stochastic variational inference.”*

Additionally in the results section:

> *“In addition to calculating median and mean absolute error, we estimate the coverage of 95% posterior latent frequencies (Supp. Fig. S8A) and posterior predictive sample frequencies (Supp. Fig. S8B) across models. We generate the posterior predictive coverage by sampling random counts for each variant using their posterior latent frequencies conditioning on the total sequences being those observed retrospectively. We find that the posterior predictive coverage is generally higher and a better fit for the models in question. Additionally, we find that the coverage is lower in countries with the highest sequencing intensity like the US and UK, suggesting that there may be over-dispersion in the sequence counts relative to binomial or multinomial sampling.”*

3 – The authors state the chose median and mean absolute error in frequency to evaluate model performance, but don’t give proper justification for why this is the correct choice of metrics. Did the authors consider other metrics often used in forecasting such as coverage deviation, weighted interval scores, brier scores, or even [Wasserstein distance] for evaluation of probability distributions?

We agree that that is essential to explain our choice of metrics in evaluation and have added the following section to our Methods section:

> *" Throughout this study, we primarily use the median and mean absolute error to evaluate the accuracy of our point forecasts. We select the median absolute error as a measure of central tendency on our forecast errors, reducing the influence of outliers and skewed data distributions due to the contribution of forecasts which tend to diverge rapidly in forecast lead. To balance this and account for the effect of outliers and rapidly divergent forecasts, we also use the mean absolute error which is less sensitive to outliers than the mean square error and has units in terms of frequencies directly. However, these are not the only possible choices for error metrics, and are motivated by our decision to focus primarily on point forecasts of variant frequencies. To supplement this analysis, we also address the coverage of probabilistic extensions of the models discussed here."*

4 – The authors necessarily have to summarize over many time points, reference data sets, countries, and variants to get some sense of overall model performance. However, given that these models are most useful to public health when novel variants are emerging with limited available sequences, it would be useful for the reader to see the results of these models applied to an emerging variant, and to be able to visually compare estimates at these critical times across models, similar to the approach taken in Susswein et al. Figure 1 could be expanded to focus in on say the emergence of a variant that ultimately took off and how the model estimate compared to the ground truth and the data available as of that time, for each reference date, for the MLR model versus a less accurate model.

We thank the reviewers for this suggestion. We focus on a period of variant emergence, showing how the designation of new lineages affects forecast accuracy of the MLR

model though we do not do a comprehensive analysis and head-to-head of models at multiple emergences. We also added a section describing the comparison of retrospective and real-time Nextclade versions and their effects on forecast accuracy:

> *"The main analyses discussed in this manuscript rely on subsetting and filtering SARS-CoV-2 sequence metadata accessed on a particular data. However, the clade designations used throughout this manuscript may not have been the same as clade designations at the time the data was available. To understand how this affects our evaluation of forecast error, we compare the accuracy of models fit to the sequence counts from metadata at the time and using the available Nextclade version to those fit on the retrospective Nextclade version used in the rest of the analyses in this paper. In particular, we focus on the timing of the designation of Omicron 22E in October 2022 and show the accuracy of MLR using the different data sets at different forecast leads. We compare the resulting MAE of these analyses between Nextclade versions in Supp. Figure (S9) and show trajectories from individual countries in Supp. Figures (S10-S17)"*

6 – It is not clear from the stated analysis of the impact of downgraded sequencing levels, that the right-truncation in the sequencing data is being properly accounted for in this analysis. Were the current sequences down-sampled relative to their overall proportion in each of the recent weeks, or did they have a constant level of sequencing (which is unrealistic, as can be seen from Fig 1A, even countries with high sequencing have pretty severe drop-off in sequencing in recent weeks). Clarification on how this was done, and modification if it didn't also take into account sequence turn-around times and right-truncation, is needed for this analysis and subsequent result to be useful to guide sequencing efforts.

Thank you for your comment, We agree that this is an important point to clarify, we downsample to a maximum weekly rate of sequencing. For each week, we sample at most N sequences in that week including all if there are fewer than N in that week, then

when generating the data sets at different times of analysis, we subset to sequences that were only available at time. This should account for right truncation and the submission delays. To that point, we have also included the following additions to clarify the “Downscaling historical sequencing effort” subsection of our methods:

> *“We then generated datasets for the same analysis dates and study period used in the previous analyses, generating 5 replicate subsampled data sets of sequences available at each analysis date for each eventual sequencing rate, location, and analysis date. Subsampling sequences per week before checking which sequences were available by the analysis date ensures that we respect the availability of sequences by submission date and submission delay in each country i.e. that countries with many sequences per week but long delays will maintain these delays.”*

7 – Lastly, the authors choice of models for comparison is somewhat limited. It appears from the methods that the MLR model used is a simple multinomial regression. However, hierarchical multinomial models, which pool information both across lineages and across countries have been developed with the intention of addressing the critical problem of making reliable estimates of variant growth advantages as variants are emerging (Susswein et al). A similar model appears to be in production within the codebase of this group. It would be a significant advancement to incorporate these models into this evaluation, focusing in particular on the ability of the model to make reliable estimates when novel variants are emerging.

We appreciate your feedback and completely agree. We have expanded our analysis to demonstrate a direct comparison between pooled hierarchical MLR and MLR across countries (new Figure 7) and additionally added two countries with lower sequencing capacity (Trinidad and Tobago and Vietnam). Across countries, we observe better performance with the hierarchical MLR for both mean and median absolute error for countries with less sequencing. We include a new subsection in our Methods titled “Comparing country-level and hierarchical short-term forecast models section”:

“In observing poor performance in initial period of variant emergence (Fig. 4), as well as poor performance in countries with less intensive genomic surveillance (Fig. 5), we conclude lack of data results in poor fitness estimates and so poor predictive performance. Joint modeling of data from multiple countries has been proposed as a way to getting improved estimates of variant growth advantages in general and also specifically improving frequency estimates in low and middle income countries. Hierarchical or joint forecast models for short-term frequency forecasts typically operate by pooling parameters between ‘groups‘ in a model. For our application, we pool the relative fitness of variants across countries, so that estimated relative fitnesses are informed by not just the observed relative fitness within a location, but also the relative fitnesses in other locations.

We compare the short-term forecast accuracy for individual models fit using MLR and this hierarchical MLR model in Figure 7. We find that overall the hierarchical MLR matches or outperforms the single country models in all locations and at all forecast lengths. Perhaps as expected the hierarchical MLR model matches MLR performance in countries with abundant data like the US and UK, while countries with less data like Trinidad and Tobago and Vietnam show a large performance advantage to hierarchical MLR.”

And in our Discussion section:

“Additionally, we implement a hierarchical variant of the model where multiple countries are fit simultaneously with a Normal prior on the relative fitness of a given variant between countries, so that $\delta_{v,g} \sim \text{Normal}(\delta_v, \sigma)$. Similar formulations of this hierarchical model have been used for SARS-CoV-2 frequency forecasts previously. [18]”

Overall, I think this is important work, and the work that was done was done in a clear and straightforward manner. I think the authors will need to acknowledge the shortcomings of the analysis in regards to the “real-time” nature of the evaluation, and should consider expanding to include uncertainty in their evaluation, and incorporate other models into their assessment. Additional supplementary figures such as the ones suggested seem essential for the reader to be made fully aware of the model performance across reference data sets and models. I recommend this article for acceptance with these revisions.

We thank the reviewer for their clear and constructive comments that surely were complementary and improved our manuscript.

Reviewer 2:

It was a joy to read this ms. The authors propose a framework to evaluate real-time forecasts of variant frequencies. They use the evolution of SARS-CoV-2 in 2022 as a case study.
They use their framework to compare models across different countries on a range of genomic surveillance intensity.

Perhaps what I was most excited about was the use of it to estimate minimum number of sequences needed per week is sufficient for accurate short-term forecasts. This is an incredible relevant question not just from a methodological standpoint but of capacity building and public health importance.

I have a few minor suggestions:

I suggest you use countries with much lower genomic surveillance effort as well, as while there are some differences between those countries they are all on higher end of effort. Meaning that the issues especially at the emergence of a new variant will be easier to miss.

We thank the reviewer for their kind words and we have decided to expand the countries being assessed to include two countries (Vietnam and Trinidad and Tobago) that demonstrate lower sequence capacity. This includes updates to Table 1, Figure 2, Figure 3, Figure 4 and Figure 5. As expected, we observe that these countries with lower capacity show weaker model performance (Figure 2). This turned out to be a very fruitful suggestion as including these countries effectively revealed the line between the forecasting models working well and no longer working to a satisfactory degree. We additionally made an addition to the manuscript in the "Genomic surveillance systems and forecast error" subsection:

> "*These results show that South Africa with ~16k sequences collected in 2022 and median of 173 sequences available from the previous 30-days yields a mean absolute +30 day forecasting error of 7.0% for the MLR model (Table 1), which is only slightly greater than the mean absolute error of 6.3% for the US with ~2.0M sequences collected in 2022 and of 5.8% for the UK with ~1.2M sequences collected in 2022. However, Vietnam with ~6k sequences collected in 2022 and median of 31 sequences available from the previous 30-delays yields a mean absolute forecasting error of 11.6% and Trinidad and Tobago with ~2.3k sequences collected in 2022 and median of 44 sequences available from the previous 30-delays yields a mean absolute forecasting error of 12.0%. This suggests that genomic surveillance systems with cadence and throughput greater than 50-100 sequences collected in the previous 30 days yield sufficient timely data to permit short-term forecasts.*"

It wasn't clear why for Australia model's performance was slightly different. Is it due to sampling efforts or it spurious? I suggest the authors explain this a little in more detail.

We thank the reviewer for pointing out this issue. We noticed convergence issues on several model runs, most prominently on the Australian data that affected our error calculations. We addressed the issue and another where forecasts were not generated for the full time period, and re-ran all models to ensure correct estimates.

Not surprisingly, the authors found that variability in forecast errors is partially driven by data limitations. And thus, as expected, the authors focus on hypothesis that error is largest for emerging variants that present a small window of time to observe dynamics and where sequence count data is often rare. So, while their testing is sound I suggest two additional steps:

1. This would no doubt be exacerbated in countries with low effort or very pulsed effort. It would be great to see that explored. So picking two or three countries with constantly low effort and or pulsed effort will be very useful

We appreciate this suggestion and have added two low sampling countries Trindad and Tobago and Vietnam whose available data at certain analysis dates can be seen in Supplementary figure 1. We also added the following to the manuscript in the “Genomic surveillance systems and forecast error” subsection:

> “*These results show that South Africa with ~16k sequences collected in 2022 and median of 173 sequences available from the previous 30-days yields a mean absolute +30 day forecasting error of 7.0% for the MLR model (Table 1), which is only slightly greater than the mean absolute error of 6.3% for the US with ~2.0M sequences collected in 2022 and of 5.8% for the UK with ~1.2M sequences collected in 2022. However, Vietnam with ~6k sequences collected in 2022 and median of 31 sequences available from the previous 30-delays yields a mean absolute forecasting error of 11.6% and Trinidad and Tobago with ~2.3k sequences collected in 2022 and median of 44 sequences available from the previous 30-delays yields a mean absolute forecasting error of 12.0%. This suggests that genomic surveillance systems with cadence and throughput*

greater than 50-100 sequences collected in the previous 30 days yield sufficient timely data to permit short-term forecasts."

2. To this regard, I would suggest to the authors to take their framework to evaluate models that are specifically pooling country data, such as https://www.medrxiv.org/content/10.1101/2023.01.02.23284123v4.full (cited in the ms) which address a lot of the issues related with backfilling and the under/ over estimation at emergence, heterogenous reporting. These pooled models while MLR, should be evaluated separately from single country models, for all the reasons and issues the authors state they are exploring.

Thank you for your suggestion. We have made an additional comparison analysis to demonstrate a direct comparison between pooled hierarchical MLR and MLR across all original countries and the added two countries with lower sequencing capacity. Across countries, we observe better performance with the hierarchical MLR for both mean and median absolute error (new Figure 7). We highlight this comparison in a new section in Results titled "Comparing country-level and hierarchical short-term forecast models":

> *"In observing poor performance in initial period of variant emergence (Fig. 4), as well as poor performance in countries with less intensive genomic surveillance (Fig. 5), we conclude lack of data results in poor fitness estimates and so poor predictive performance. Joint modeling of data from multiple countries has been proposed as a way to getting improved estimates of variant growth advantages in general and also specifically improving frequency estimates in low and middle income countries. Hierarchical or joint forecast models for short-term frequency forecasts typically operate by pooling parameters between 'groups' in a model. For our application, we pool the relative fitness of variants across countries, so that estimated relative fitnesses are informed by not just the observed relative fitness within a location, but also the relative fitnesses in other locations.*
>
> *We compare the short-term forecast accuracy for individual models fit using MLR and this hierarchical MLR model in Figure 7. We find that overall the hierarchical*

MLR matches or outperforms the single country models in all locations and at all forecast lengths. Perhaps as expected the hierarchical MLR model matches MLR performance in countries with abundant data like the US and UK, while countries with less data like Trinidad and Tobago and Vietnam show a large performance advantage to hierarchical MLR."

While I agree with the statements that MLR models, "do not account for future mutations and can only project forwards from circulating viral diversity. This intrinsically limits the effective forecasting horizon achievable by these models." It seems to me that the discussion should center in the purpose of the models to begin with. So, for a more balanced discussion, I would suggest the authors to add perhaps a paragraph in the discussion on the role of adding mechanism or background information (vaccine, previous waves, to understand immunity profiles of populations) onto these models, if they are to be useful beyond the nowcast, as it will no doubt affect internal dynamics and competition between variants that in turn affects sustained growth and displacement of other less fit variants.

We agree that the discussion could focus more on the purpose of these models i.e. short-term forecasts of variant frequencies. To address this, we have added the following sentences to our Discussion:

" Although these models appear largely accurate for short-term forecasts, they may be improved by incorporating underlying biological mechanism. In general, the methods discussed here are primarily statistical in nature and do not account for much of the biological or immunological knowledge that we have or could obtain. The incorporation of such knowledge could increase the short-term and medium-term capabilities of these models. Additionally, these fitness models do not account for future mutations and can only project forward from circulating viral diversity. "
