# Supplementary material for: Fitness models provide accurate short-term forecasts of SARS-CoV-2 variant frequency
Source: PLoS Comput Biol. 2024 Sep 6;20(9):e1012443. doi: 10.1371/journal.pcbi.1012443 (PMC11410224; doi:10.1371/journal.pcbi.1012443)
Supplement: Attachment — Submitted filename: #ncov-forecasting-fit reviewer response round 2.pdf [file pcbi.1012443.s020.pdf]

Fitness models provide accurate short-term forecasts of SARS-CoV-2 variant frequency

PLOS Computational Biology Response Letter

Dear Editor,

We appreciate your expertise and time in reviewing our paper and providing valuable comments. It was your valuable and insightful comments that led to possible improvements in the current version. We have carefully considered the comments and tried our best to address each of them. Below, we've included point-by-point responses to reviewer critiques.

Sincerely,

Eslam Abousamra, Marlin Figgins, and Trevor Bedford

Reviewer #1: Overall, the manuscript is of great importance to public health and specifically estimation and evaluation of variant dynamics. The authors made significant improvements to the manuscript in the course of their revisions, and I would highly recommend this article for publication in PLOS Computational Biology, contingent on a few minor revisions/suggestions:

Revisions:
- Suggested edit in methods titled "Comparing forecasts using retrospective clade designations and real-time designations" -> "The main analysis discussed in…. accessed on a particular date" (date instead of what is written now which is data)

Thank you for pointing out this typo. We've corrected this in the manuscript.

- Suggest clarifying terminology, when referring to Nextclade version to indicate that this means the clade designation model. It might be confusing to a reader who is already thinking about time-stamped versions of the data to distinguish between the two, but this distinction is critical to understanding the difference between the real-time and retrospective variant calling.

This is indeed tricky. We've tried to clarify by updating the relevant manuscript text as follows:

> "To understand how this affects our evaluation of forecast error, we compare the accuracy of models fit to the sequence counts from metadata at the time and using the available Nextclade reference tree to those fit on the retrospective Nextclade reference tree used in the rest of the analyses in this paper. This compares lineage designations that were available in real-time on the historical analysis date to lineage designations that are retrospectively available."

- I would suggest calling a variant by its pangolin with Nextclade beside it throughout e.g. BQ.1 (Omicron 22E)

We've gone through the manuscript and have been sure to have every reference to a Nextstrain clade include the corresponding Pango lineage, eg

> "Consistent with less available data, we observe that the model predictions for Japan were more frequently misestimated compared to the United States with particularly large differences for clades 22B (lineage BA.5) and 22E (lineage BQ.1)..."

Suggestions:
- I would clarify in the description of the downsampling, that we might expect that when sequencing declines overall, it would also decline in recent weeks, instead of the recent weeks being the upper limit, and so the analysis may not reflect the real-time impact of reducing sequencing quantity unless the speed of sequencing is prioritized.

We appreciate the reviewer's comment here and have further clarified the text for the downscaling analysis. We now explicitly mention that by maintaining the existing submission delays in this analysis, we replicate the decline in available sequences in recent weeks.

> "Subsampling sequences per week before checking which sequences were available by the analysis date ensures that we respect the availability of sequences by submission date and submission delay in each country, so that countries with many sequences per week but long delays will maintain these delays. Therefore, the selected sequencing rate sets an upper limit on the number of sequences available per week at any analysis date and preserves the decline in available sequences that we typically observe in recent weeks since we only include sequences which are within our original subsample and available at the time of analysis."

- I’m not sure why the hierarchical MLR results are not included in Figures 2 and 3, and instead are an add on in Figure 7. The manuscript might read more smoothly if the hierarchical MLR is presented alongside the other models including the single country MLR. Right now it appears like a secondary analysis.

We appreciate the reviewer’s perspective here, but would like to keep the current manuscript structure with initial analysis comparing country-specific MLR and variant Rt models (Figures 2 and 3) and then in a secondary analysis compare hierarchical to non-hierarchical MLR (Figure 7). We believe that these are effectively looking at two different things. In the first case, it’s how well MLR and other models work on exactly the data at hand. In the second case, we can use standard statistical pooling logic to improve estimates, but it’s a separate concept. We also feel that in manuscript flow, it makes sense to first present the issue of countries with less developed genomic surveillance having less accurate forecasts (Figure 5) before presenting the (partial) solution of hierarchical models (Figure 7).

- I would suggest that the authors describe in further detail why they think the coverage is so low in high sequencing countries? Is this because the model is underdispersed/over confident? If so, could they offer any possible solutions to this problem in the discussion (e.g. a different observation model, models accounting for heterogeneity in facilities, etc.)

This is indeed an important limitation in the current MLR models. We believe this is due to a combination of over-dispersion of sequence counts alongside evolution impacting clade-specific growth advantage through time. We’ve included a couple additional sentences in Results:

> “We also observe that coverage is higher for the GARW model that allows for time-varying growth advantage than for the FGA or MLR models which enforce a fixed growth advantage. As clades evolve and new subclades emerge we expect clade-specific growth advantage to change alongside.”

We've included an additional paragraph in the Discussion to present possible solutions in future work:

> "However, we observe that coverage is generally lower than ideal with predictive coverage under 50\% for countries with the most sequencing (Supp. Fig. S8B). We believe this may be due to a combination of over-dispersion of sequence counts relative to the multinomial sampling assumption as well as clade-level growth advantages changing through time as clades evolve. The former could be addressed by including over-dispersion in the sequence observation model and the latter could be addressed by implementing growth advantages that vary through time in an auto-correlated fashion."

- Figures S10-17 are a bit confusing, which lines represent the fit to the real time next clade and which lines are fit to the current nextclade? As presented, there appears to only be one model fit, it would be helpful to present the model fit to both input data sets to compare accuracy to both the real time and retrospective assignments. In general, [I] like these figures though because they make clear some examples of the forecasts versus the later observations.

We've updated these supplemental figures to clarify what is happening in these plots. This includes clearly marking that the frequencies in each panel correspond to two different data sets and model fits. The first row (A) shows the fit of the models to the retrospective clade designations. The second row (B) shows the fit of the models to the data using the clade designations available at the time for the same analysis dates in the first row. We've also updated these figures to use both the Nextstrain clade name as well as the lineage name.

Reviewer #3: In the article: "Fitness models provide accurate short-term forecasts of SARS-CoV-2 variant frequency" the authors provide a nice demonstration of the performance of different variant forecasting models for SARS-CoV-2 variants and

provide estimates for the sample size necessary for countries surveillance programs. The article is well written and covers an important topic, particularly as the US is considering a new variant forecasting competition. Based on their revisions to reviewer feedback I believe this article should be accepted for publication, but I have a single suggestion the authors should consider adding.

Suggestion: The authors provide a sample size that they feel would be sufficient to have reasonable data for stable variant forecasting based on a nice downsampling analysis. One issue is that many countries are not currently reporting 1,000 cases a week, some not even 200! It would be useful if the authors could try and speak to these countries regarding the genomic sequence sample sizes necessary. My feeling is that it should be reasonable to carry out nowcast/forecasts in a scenario where there are 200 reported cases a week and all of them are sequenced, though I think it's an interesting question. It would be great if the authors could analyze the fraction of reported cases being sequenced in their sample size analysis (maybe some simple back-of-the-envelope calculations could be made) to see if they can provide any guidance in these scenarios where there are smaller reported case counts.

This is an excellent point and something we haven't at in detail. However, since 2022, there has been a large disconnect between reported case counts and viral circulation. For example, Afghanistan with a population of 41M reported 638 cases in epiweek 20 of 2024, while Brunei with a population of 500k reported 175 cases in this same time period. This is 1.5 cases per 100k in Afghanistan and 35 cases per 100k in Brunei. This 23-fold difference is going to be almost entirely reporting rate differences. Similarly, South Korea has 135,331 cases reported for epiweek 20 of 2024. This is 265 cases per 100k and so a 176-fold difference in reporting rate relative to Afghanistan.

We believe that population size is going to be a much more reliable indicator of absolute viral prevalence than reported cases. We expect that absolute viral prevalence should indeed matter for variant frequency dynamics. However, we observed that for Denmark

with 6M people we weren't yet seeming to reach the point at which stochastic effects start to manifest.

We've added a paragraph to the Discussion going over these issues, but focusing on viral absolute prevalence and how it relates to fitness model predictions:

> "In addition to differences in genomic surveillance, we expect countries may differ in variant dynamics due to differences in absolute viral prevalence. We expect that variant frequencies we more closely follow the MLR expectation when absolute prevalence is large, achieved through a large host population and/or frequent repeated infection. Rapid continued evolution of SARS-CoV-2 [19] suggests that we will continue to see widespread circulation of SARS-CoV-2 and thus we generally expect fitness-based models to provide an adequate description of variant frequency dynamics. Of note, in comparing the UK with 67M people to Denmark with 6M people we observe similar levels of prediction error when sampling sequences at similar intensities (Fig. 6). This suggests that stochastic effects from low absolute viral prevalence were not strongly manifesting with a population size of 6M. However, we do expect that at some smaller population size stochastic effects and repeated importations will cause a deviation in frequency dynamics from fitness model expectations."

In additional, given interest in these pragmatic sample size results, we've included an additional paragraph in the Discussion that attempts to define minimum thresholds:

> "In live MLR analyses at nextstrain.org/sars-cov-2/forecasts/, we have relied on the number of sequences available from samples collected in the previous 30-days as the key metric for inclusion of a country in the analysis. Along these lines, for pragmatic guidance for thresholds in which to trust MLR results, we observe that Trinidad and Tobago with 2.3k sequences collected in 2022 and a median 30-day sequence count of 43 shows a mean absolute forecasting error of 12%, that Vietnam with 6k sequences collected in 2022 and a median 30-day

sequence count of 30 shows a mean absolute forecasting error of 11% and that South Africa with 16k sequences collected in 2022 and a median 30-day sequence count of 170 shows a mean absolute forecasting error of 7%. This suggests that a threshold of 50 sequences in previous 30 days should be roughly consistent with a $\sim$10\% forecasting error. Keeping forecasting error under 10% seems like a reasonable target for public display of frequency forecasts and would support targeting a threshold of 50 sequences from samples collected in the previous 30 days.”
