# Supplementary material for: Fitness models provide accurate short-term forecasts of SARS-CoV-2 variant frequency
Source: PLoS Comput Biol. 2024 Sep 6;20(9):e1012443. doi: 10.1371/journal.pcbi.1012443 (PMC11410224; doi:10.1371/journal.pcbi.1012443)
Supplement: Attachment — Submitted filename: PCOMPBIOL-D-23-01974 Review.docx [file pcbi.1012443.s018.docx]

PLOS CB Review of Fitness Eval Models paper

Overview of the paper:

In this manuscript, the authors perform a retrospective analysis to compare the performance of 4 different models aimed at nowcasting and forecasting variant frequency. They construct reference data sets to mimic the SARS-CoV-2 sequences that would have been available in GISAID at each reference date in 6 different countries, and apply a single Nextstrain clade assignment model across all reference datasets. The Nextstrain clade assignment categorizes each sequence into one of 7 clade assignments that correspond to the circulating and emerging lineages in 2022: BA.1, BA.2, BA.4, BA.5, BA.2.12.1, BA.2.75, and BQ.1. They then pass these datasets of sequence counts by collection date into each of the 4 models and a baseline “naïve” model for comparison, for each country and each reference date. They assess the performance of each model using median and mean absolute error in the variant frequency estimate of each model, where ground truth is defined as the 7-day rolling averaged variant proportion from a later reference date (when presumably all/most sequences collected on those days had been submitted). They average model performance across all variants and reference time points and examine how model performance varies from 30 days before the forecast time (nowcast) to 30 days ahead of the forecast time. As a secondary analysis, they investigate the relationship between sequence quantity and quality on model performance, both empirically (by comparing across the countries with differences in sequencing characteristics) and through simulation. They generate simulated datasets with reduced sequencing levels by randomly sampling from the available sequences to obtain a constant number of sequences per week. They then refit the models to each of the datasets at the different sequencing levels and compare the average performance of the models.

The authors acknowledge that while the multinomial logistic regression (MLR) model performs “well” when averaging over all variants and reference time points, a subset of predictions have very large error. They hypothesize that this error is largest when variants are emerging and there are comparably few sequences of that variant.

My review:

The authors have addressed a very critical problem in the field of public health, particularly relevant in the wake of an explosion in the availability of genomic sequencing and its use for public health decision-making for COVID-19. While a number of models have been developed, and a number of estimates of variant frequencies are made public, there is comparably little rigorous evaluation of the nowcasts and forecasts of these model outputs. One of the reasons evaluation is less straightforward than evaluation of forecasts of other epidemiological indicators such as hospital admissions, cases, or deaths, is that the definition of the quantity of interest itself is also dynamic – the upstream models used to assign a sequence to a particular lineage are themselves constantly changing. Acknowledging this difficulty and complexity of the problem the authors tried to tackle, I still have a number of reservations about the work:

- The authors describe how they attempted to reconstruct datasets that would have been available as of each reference date, using only sequences that had been submitted by a certain date. This made sense, however, it seems that they applied the same Nextstrain clade assignment model across all of the reference datasets. If the Nextstrain clade assignment model is at all similar to pangolin assignment model, the assignments are not stable over time. Therefore, to do this kind of evaluation properly you would need to have run the clade assignment using the Nextstrain clade assignment model available at the time that the estimate was made, and then compare it to the observed proportions based on the updated clade assignment model, because this represents what you would have forecasted with the information available at the time. For this reason, I don’t think the authors can claim that this analysis is truly “real-time” evaluation, but I do understand why they made this choice as it makes for a clean comparison if purely concerned with model performance given a stable set of lineage assignment information.
- The authors did not include any incorporation of the uncertainty of estimates into their evaluation. Given the stated hypothesis that models perform worse when there is limited data about emerging variants, it feels critical to evaluate model estimates with uncertainty, rewarding models who properly quantify the uncertainty at those critical times. Even if not every model returns a full distribution of estimates, they could at the least do an analysis using the confidence intervals of the estimates and assessing the coverage of their intervals compared to the “truth” data
- The authors state the chose median and mean absolute error in frequency to evaluate model performance, but don’t give proper justification for why this is the correct choice of metrics. Did the authors consider other metrics often used in forecasting such as coverage deviation, weighted interval scores, brier scores, or even [Wasserstein distance](https://en.wikipedia.org/wiki/Wasserstein_metric) for evaluation of probability distributions?
- The authors necessarily have to summarize over many time points, reference data sets, countries, and variants to get some sense of overall model performance. However, given that these models are most useful to public health when novel variants are emerging with limited available sequences, it would be useful for the reader to see the results of these models applied to an emerging variant, and to be able to visually compare estimates at these critical times across models, similar to the approach taken in [Susswein et al](https://www.medrxiv.org/content/10.1101/2023.01.02.23284123v4.full). Figure 1 could be expanded to focus in on say the emergence of a variant that ultimately took off and how the model estimate compared to the ground truth and the data available as of that time, for each reference date, for the MLR model versus a less accurate model.
- To expand on the previous point, there seems to be missing supplementary figures/analysis that demonstrate the performance of all 4 of the models being assessed compared to the retrospective truth data shown in Figure 1.
- It is not clear from the stated analysis of the impact of downgraded sequencing levels, that the right-truncation in the sequencing data is being properly accounted for in this analysis. Were the current sequences down-sampled relative to their overall proportion in each of the recent weeks, or did they have a constant level of sequencing (which is unrealistic, as can be seen from Fig 1A, even countries with high sequencing have pretty severe drop-off in sequencing in recent weeks). Clarification on how this was done, and modification if it didn’t also take into account sequence turn-around times and right-truncation, is needed for this analysis and subsequent result to be useful to guide sequencing efforts.
- Lastly, the authors choice of models for comparison is somewhat limited. It appears from the methos that the MLR model used is a simple multinomial regression. However, hierarchical multinomial models, which pool information both across lineages and across countries have been developed with the intention of addressing the critical problem of making reliable estimates of variant growth advantages as variants are emerging ([Susswein et al](https://www.medrxiv.org/content/10.1101/2023.01.02.23284123v4.full)). A similar model appears to be in [production](https://nextstrain.github.io/forecasts-ncov/) within the [codebase](https://github.com/blab/evofr/blob/main/evofr/models/mlr_hierarchical.py) of this group. It would be a significant advancement to incorporate these models into this evaluation, focusing in particular on the ability of the model to make reliable estimates when novel variants are emerging.

Overall, I think this is important work, and the work that was done was done in a clear and straightforward manner. I think the authors will need to acknowledge the shortcomings of the analysis in regards to the “real-time” nature of the evaluation, and should consider expanding to include uncertainty in their evaluation, and incorporate other models into their assessment. Additional supplementary figures such as the ones suggested seem essential for the reader to be made fully aware of the model performance across reference data sets and models. I recommend this article for acceptance with these revisions.
